# Methods of Manipulation of Acoustic Radiation Using Metamaterials with a Focus on Polymers: Design and Mechanism Insights

**DOI:** 10.3390/polym16172405

**Published:** 2024-08-24

**Authors:** Qibo Deng, Tianying Du, Hassanien Gomaa, Yong Cheng, Cuihua An

**Affiliations:** 1 School of Mechanical Engineering, Hebei University of Technology, Tianjin 300401, China; duty1202@163.com (T.D.); h.gomaa@azhar.edu.eg (H.G.); ancuihua@hebut.edu.cn (C.A.); 2 Department of Chemistry, Faculty of Science, Al-Azhar University, Assiut 71524, Egypt; 3 Hebei Key Laboratory of Mechanical Reliability for Heavy Equipments and Large Structures, Yanshan University, Qinhuangdao 066004, China

**Keywords:** acoustic manipulation, coordinate transformation methods, acoustic metamaterials, polymers

## Abstract

The manipulation of acoustic waves is becoming increasingly crucial in research and practical applications. The coordinate transformation methods and acoustic metamaterials represent two significant areas of study that offer innovative strategies for precise acoustic wave control. This review highlights the applications of these methods in acoustic wave manipulation and examines their synergistic effects. We present the fundamental concepts of the coordinate transformation methods and their primary techniques for modulating electromagnetic and acoustic waves. Following this, we deeply study the principle of acoustic metamaterials, with particular emphasis on the superior acoustic properties of polymers. Moreover, the polymers have the characteristics of design flexibility and a light weight, which shows significant advantages in the preparation of acoustic metamaterials. The current research on the manipulation of various acoustic characteristics is reviewed. Furthermore, the paper discusses the combined use of the coordinate transformation methods and polymer acoustic metamaterials, emphasizing their complementary nature. Finally, this article envisions future research directions and challenges in acoustic wave manipulation, considering further technological progress and polymers’ application potential. These efforts aim to unlock new possibilities and foster innovative ideas in the field.

## 1. Introduction

Sound waves are omnipresent in our daily lives, and with the passage of time and the development of science and technology, the range of applications of sound waves in human production and daily life is increasing. The central issue in the application of sound waves is the ability to regulate them at will, which has attracted the attention of numerous researchers [1,2,3,4,5,6,7,8,9,10,11]. The ability to precisely control and guide the propagation and shape of acoustic waves is critical for developing high-performance acoustic devices, acoustic imaging, acoustic communications, and more. Research is flourishing, particularly in the underwater and medical sectors [12,13,14,15,16,17,18,19]. In the field of underwater technology, researchers strive to advance the performance and dependability of underwater cloaks and design groundbreaking sonar technologies to increase the effectiveness of oceanic exploration and the exploitation of marine resources. In medicine, researchers are pursuing innovative ultrasound imaging techniques and treatments to improve medical imaging accuracy and effectiveness. Acoustic manipulation holds tremendous importance in medicine and underwater research, promising numerous benefits and innovative applications for humanity.

Researchers continuously investigate novel manipulation techniques and materials to achieve precise control of acoustic waves. The application environment of sound waves is complex and changeable. When the sound wave is propagating, the acoustic impedance of different environmental media does not match, which will cause the attenuation of the sound wave. The acoustic impedance is defined as $Z=\sqrt{\rho \kappa }$, where Z is the acoustic impedance, ρ is the medium density, and κ is the medium bulk modulus. The acoustic impedance range of typical liquids (such as water, ethanol, seawater, sulfuric acid, etc.) is $0.81\times 10^{6}-2.34\times 10^{6}\;\mathrm{Pa}\;s/m$, the acoustic impedance range of typical gases (such as air, oxygen, nitrogen, etc.) is $0.41\times 10^{3}-0.51\times 10^{3}\;\mathrm{Pa}\;s/m$, and the acoustic impedance range of typical soft solid polymers (such as rubber, polyethylene, polystyrene, etc.) is $1.39\times 10^{6}-2.41\times 10^{6}\;\mathrm{Pa}\;s/m$ [20]. Two prominent avenues of research in acoustic wave manipulation are the coordinate transformation methods and acoustic metamaterials. These research avenues have garnered significant attention for achieving valuable acoustic wave manipulation. The coordinate transformation technique, a crucial process for modulating acoustic waves, was originally used to modulate electromagnetic waves. It has since been adapted for acoustic wave manipulation because of similarities in the equations governing fluctuations of electromagnetic and acoustic waves. This technological tool has increased the flexibility and controllability of acoustic research and applications. It has facilitated the development of acoustic technology in communications, medical imaging, environmental monitoring, sound processing, and materials research. Through a comprehensive study and application of the coordinate transformation methods, we aim to unlock their untapped potential and provide new opportunities for innovation and progress in the field of acoustics. Complementary to the coordinate transformation methods is the investigation of metamaterials. Polymers play an important role in the field of acoustic metamaterials. Polymers can be fabricated into key components of acoustic metamaterials to control and adjust the propagation characteristics of sound waves. Researchers are exploring the application potential of different types of polymers in acoustic metamaterials [21,22]. Rubber materials have high stretchability, wide application scenarios, and good sound absorption performance [23]. Polyethylene (PE) has a low cost and excellent sound insulation effect [24]. Polylactic acid (PLA) has good biocompatibility and low density. It can be used to prepare medical acoustic equipment and lightweight acoustic metamaterials [25]. Polydimethylsiloxane (PDMS) is soft, heat resistant, and stable for precise acoustic control and sensing applications [26]. Polymers have their own advantages in acoustic metamaterials, which can flexibly design and optimize structures according to specific acoustic requirements, and achieve a balance between environmental protection and cost-effectiveness. The dielectric constant (*ε*) and the magnetic permeability (*μ*) are fundamental physical quantities used in the propagation of electromagnetic waves, and there is no way for matter in nature to achieve a negative value for both simultaneously. In 1968, Veselago et al. deduced that the propagation of electromagnetic waves in any phase would occur when *ε* < 0 and *μ* < 0. This finding inspired and stimulated the advancement of electromagnetic metamaterials [27]. In 2006, D. Schurig discovered a copper cylinder concealed in a cloak constructed using a coordinate transformation. The cloak diminishes the scattering of the concealed object while decreasing its shadows, creating empty space when the object and cloak are paired [28]. Acoustic metamaterials are artificial structures that can control, guide, and modulate the propagation of acoustic waves and the shape of the wavefront. They can achieve these effects through specially designed material and structural parameters. By adjusting subtle structural and material parameters, acoustic metamaterials achieve specific manipulation effects on sound waves’ refraction, transmission, and reflection [29,30,31,32,33,34,35,36]. In 2000, Z.Y. Liu et al. developed acoustic metamaterials capable of expressing the negative density characteristics of materials. This results in phase change or phase reversal when sound waves propagate through the negative density area. This unique behavior allows the law of reflection to be violated and acoustic waves to propagate in the reverse phase [37]. Acoustic metamaterials can also achieve negative modulus properties of materials. In 2006, Nicholas Fang designed acoustic metamaterials that can achieve strong dispersive properties of acoustic waves. These materials can realize phase velocities that are antiparallel to the group velocities when acoustic waves are transmitted [38]. On the other hand, an acoustic metasurface is often regarded as two-dimensional correspondences of metamaterials. The acoustic metasurface changes the reflection and transmission characteristics of the acoustic wave at the interface by designing the microstructure and modifying the material parameters, so as to achieve accurate control of the acoustic wave front [39,40,41,42,43,44]. In 2011, N.F. Yu et al. introduced the concepts of generalized Sneer’s law and hypersurfaces. These concepts focus on designing an artificial structure at the interface of two different media to create an abrupt phase shift, ultimately achieving a phase discontinuity. This method offers immense flexibility for wave control [45]. Acoustic metamaterials have many applications in acoustic wave manipulation and can be designed in conjunction with other technological methods, such as coordinate transformations. In 2006, J.B. Pendry and others used a combination of metamaterials and coordinate transformations to achieve electromagnetic stealth by hiding an object in a given volume of space [46]. U. Leonhardt proposed that all parallel beams of incident light would bend around a hole and converge in the same direction as they entered the medium and suggested that a space other than the original physical space could achieve perfect invisibility for any object placed in the hole [47]. Alternatively, acoustic metamaterials can be combined with topology optimization, allowing specific acoustic functions such as beam focusing and optimal waveguide transmission modes to be achieved by varying parameters such as material distribution, shape, and pore structure. Ultimately, this opens up new possibilities for high-performance acoustic devices and applications [48,49,50,51,52,53].

This review concentrates on the coordinate transformation methods and acoustic metamaterials. It introduces the application of the coordinate transformation methods to acoustic wave manipulation, encompassing multi-regional division of objects and medium manipulation. Additionally, it reviews the research progress of polymer acoustic metamaterials in modulating acoustic wave frequency, phase and scattering, and their wide practical applications are introduced. Furthermore, this paper discusses the combined application of coordinate transformation methods and acoustic metamaterials, and points out the future research directions and challenges, aiming to promote the development of acoustic manipulation technology and its practical application.

## 2. Coordinate Transformation Methods for Wave Variation and Conditioning

The coordinate transformation methods are first applied to the manipulation of electromagnetic waves, and then extended to the manipulation of acoustic waves. An electromagnetic wave is essentially a transverse vector wave, while an acoustic wave is usually a longitudinal scalar wave. However, the wave equations of electromagnetic waves and acoustic waves can be expressed in a similar mathematical form. This mathematical versatility makes the manipulation of electromagnetic waves and sound waves have some theoretical similarities. However, there are obvious differences in specific signal processing. For example, the phase of an electromagnetic wave is affected by the oscillation of the electric field and magnetic field. Acoustic waves are affected by the propagation medium. These differences lead to different physical behaviors in applications such as propagation and imaging. The following section describes the use of the coordinate transformation technique for controlling electromagnetic and acoustic waves and how acoustic waves are controlled using this method by means of two techniques: multi-region division and medium control.

### 2.1. Electromagnetic Waves

**Figure 1:**
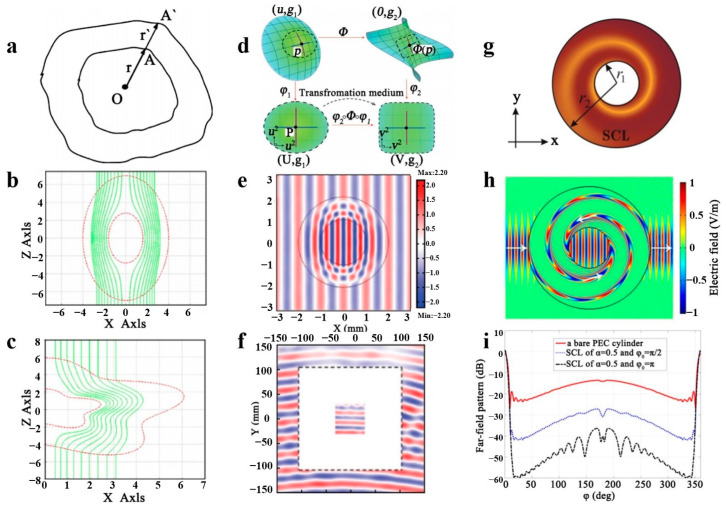
A coordinate transformation method is proposed for regulating electromagnetic waves. Based on a geometric coordinate transformation method (**a**), an ellipsoidal electromagnetic cloak (**b**) and an arbitrary geometric cloak (**c**) are designed. Reproduced with permission from [58]. Copyright © 2021, Elsevier. The electromagnetic wave concentrator is designed based on a coordinate transformation process (**d**) and the simulation results (**e**,**f**). Reproduced with permission from [59]. Copyright © 2019, Wiley. An electromagnetic cloak designed based on a coordinate change method (**g**) and a preset electric field and power trend (**h**), and radar detection results (**i**). Reproduced with permission from [60]. Copyright © 2019, AIP Publishing.

Transform optics offers a novel method for regulating electromagnetic waves in any desired way [54]. In recent years, much research has combined transformation optics with metamaterials, revealing new phenomena such as frequency conversion [55], dispersion engineering [56], and bandwidth extension. S.C. Tao and H.Y. Chen examined the crucial influence of perfectly matched layers on electromagnetism [57]. They devised an integrated, perfectly matched layer through coordinate transformation theory composed of material parameters from a radial coordinate transformation of a complex plane. After a comparative analysis, it was found that the integrated, perfectly matched layer provides the most effective absorption without backscattering. Therefore, it can serve as an absorbing core for electromagnetic simulations and related experiments. The methodology for coordinate transformation, as posited by H.H. Sidhwa et al. [58], is depicted in Figure 1a. The initial stage encompasses the creation of a symmetrical elliptical-shaped electromagnetic cloak, while the subsequent phase is concerned with its extension to a cloak of arbitrary convex geometry, as illustrated in Figure 1b,c. However, L. Xu and H.Y. Chen [59] employed coordinate transformations to offer a geometric procedure to manipulate electromagnetic waves at will, as revealed in Figure 1d. Mathematical coordinate transformations that yield distortions from the initial space U can be efficiently implemented into a transformed medium with non-uniformity in space V. By utilizing this transformation, a concentrator can be designed, as illustrated in Figure 1e. By employing the structural arrangement, one can acquire measurements at a specific resonant frequency, as shown in Figure 1f. Note that the white region depicts the space inaccessible to the detector antenna. M. H. Fakheri and A. Abdolali designed a swamp coating (SCL) using complex coordinate transformations. They proposed a new strategy to reduce the radar cross-section (RCSR) of two-dimensional targets of arbitrary shape, which can simultaneously control the amplitude and phase of electromagnetic waves [60]. Figure 1g displays the SCL shaded area and air represented by a white area. By altering the relationship, the electromagnetic waves can propagate along an Archimedean spiral, as demonstrated in Figure 1h. The explanation of the radar interface involves testing different SCL coatings on a perfectly conducting cylinder, represented in Figure 1i. It was discovered that these coatings cause the incident wave to dissipate, ultimately resulting in RCSR. J.W. Xu presented a coordinate transformation method that proficiently handles constructions with intricate laminations [61]. This technique regulates the electromagnetic field via object multi-region division and medium manipulation. These concepts were initially employed in the realm of electromagnetic waves and later expanded to encompass other forms of wave dynamics, including elastic waves, acoustic waves, surface water waves, and even static fields [62,63].

Early related research dates back to the problem of the Helmholtz equations in the late 19th century. J.C. Maxwell proposed a set of equations describing electromagnetic phenomena in the mid-19th century, now known as Maxwell’s equations. These equations were developed using the method of coordinate transformation to derive them. Applying the coordinate transformation method to Maxwell’s work enabled him to theoretically investigate the existence and propagation of electromagnetic waves. Maxwell advanced the theory of electromagnetism to a new level and established the foundation for studying electromagnetic waves by utilizing the coordinate transformation technique to analyze and derive equations.

The equations governing electromagnetic and acoustic waves have certain similarities, especially in formulating their fluctuation equations. For electromagnetic waves, their fluctuation equations are derived from Maxwell’s system of equations. Under vacuum conditions, the fluctuation equations of the electromagnetic field can be expressed as follows:

(1) $$\nabla^{2}E-n^{2}\mu_{0}\varepsilon_{0}\;\partial^{2}E/\partial t^{2}=0$$

(2) $$\nabla^{2}E-n^{2}\mu_{0}\varepsilon_{0}\;\partial^{2}B/\partial t^{2}=0$$

where ∇^2^ represents the Laplace operator, *E* represents the electric field, *B* represents the magnetic field, *n* denotes the refractive index of the medium, *μ*_0_ represents the magnetic permeability, *ε*_0_ represents the dielectric constant, and *t* represents time. This equation demonstrates the connection between the second-order spatial gradient of the electromagnetic field and the second-order derivative concerning time.

For sound waves, the pressure change can be described using the following equation for fluctuations:

(3) $$\nabla^{2}p-(1/c^{2})\partial^{2}p/\partial t^{2}=0$$

where ∇^2^ denotes the Laplace operator, *p* represents the variation in the pressure wave, *c* represents the speed of sound, and *t* represents time. Furthermore, this equation demonstrates the correlation between the second-order spatial gradient of pressure and the second-order derivative of time.

Although these two equations may appear dissimilar, their form is quite similar. Both are second-order partial differential equations describing fluctuations’ propagation through space. They possess comparable attributes of fluctuations, including propagation speed, wavelength, frequency, and many more. Therefore, the introduction of the coordinate transformation methods have extended to the research domain of acoustic waves. By establishing a suitable coordinate system and utilizing appropriate coordinate transformation techniques, complex acoustic phenomena can be simplified into a model that is easier to comprehend and examine. This results in the original complex equations being reduced to a more manageable form, thereby enabling the development of specific manipulation strategies and schemes for acoustic waves.

### 2.2. Sound Waves

Implementing the coordinate transformation methods enables the division of objects into multiple regions and the conditioning of acoustic waves within the medium. It should be emphasized that the conditioning and processing of acoustic waves using coordinate transformations is performed based on appropriate physical assumptions and applicable conditions. The selection of a proper coordinate transformation method necessitates careful attention to the characteristics and objectives of the acoustic wave problem, as well as a rigorous mathematical analysis for determining the optimal approach. Typically, coordinate transformations are implemented by simplifying the model or approximating the treatment without altering the inherent acoustic properties of the medium.

#### 2.2.1. Multi-Region Division of Objects

The coordinate transformation methods are applicable to the multi-region classification of objects in sound waves. With suitable coordinate transformations, an object’s region or structural shape can be divided, thus impacting sound wave propagation and reflection. Multi-region object classification involves operations such as splitting and scaling geometrical shapes, thereby altering the method for describing and solving acoustic wave problems. During such transformations, coordinate transformations can aid in simplifying the problem’s form, decreasing computational complexity, and improving comprehension of the physics involved. This process is particularly significant in analyzing and designing acoustic devices and barriers, among other applications.

**Figure 2:**
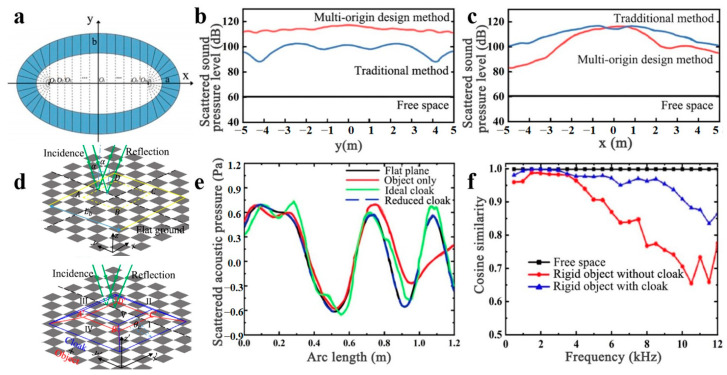
(**a**) The coordinate transformation method of elliptical acoustic cloak. The sound pressure scattering effect of the acoustic cloak when the sound wave is incident in the *x*-axis (**b**) and *y*-axis (**c**) directions. Reproduced with permission from [64]. Copyright © 2022, Springer Nature. Based on the multi-region segmentation coordinate transformation (**d**), the scattering pressure field (**e**) and cosine similarity simulation (**f**) of the acoustic cloak are designed. Reproduced with permission from [65]. Copyright © 2019, IOP Publishing.

The regulation of incident waves in various directions can be efficiently achieved using the multi-area division coordinate transformation. By utilizing area division and multi-origin coordinate transformation, an acoustic cloak of arbitrary shape in two dimensions can be realized [64], and the functionality of the designed acoustic cloak in diverse wave incident directions can be delineated. The researchers studied the adjustment effect of the elliptical cylindrical cloak under different incident wave directions, and the transformation method is shown in Figure 2a. The researchers compared the traditional elliptic coordinate transformation with the new multi-origin coordinate transformation. The simulation results are shown in Figure 2b,c. The novel multi-origin design approach has the potential to significantly alleviate the issue of the poor regulation of small-axis incident acoustic waves in the conventional elliptic cylindrical cloak. The simulation results confirm the proposed design scheme’s feasibility and effectiveness. They also broaden the scope of the coordinate transformation-based acoustic cloak design, particularly for intricate regions that can be decomposed into basic shapes, and the incoming wave direction is known beforehand. Furthermore, the framework’s stealth effect of the three-dimensional acoustic cloak is accomplishable via the multi-region coordinate transformation approach [65]. As illustrated in Figure 2d, four points, namely A, B, C, and D, located on the ground in the z direction undergo simultaneous compression in virtual space and are transformed into corresponding points A’, B’, C’, and D’ in physical space. This creates a void space on the ground that can be used to hide objects, while the transformed/compressed space can redirect the incident wave in virtual space. The virtual and physical spaces are systematically mapped through linear transformation to establish a point-to-point relationship. The coordinate transformation method transforms the corresponding material parameters in the acoustic cloak shell. This establishes the material parameters of the cloak in each region to the mapping function. Good invisibility performance is demonstrated in a wide frequency range, as illustrated in Figure 2e,f. However, the composite material suggested by the researcher to create the acoustic stealth cloak is mercury and further thought is needed regarding its safety for practical implementation. Coordinate transformations can attain external acoustic stealth without fully enclosing a target [66]. The proposed device’s stealth effect remains unchanged, regardless of the target’s intrinsic material or shape. This allows the target to move within the concealed area while retaining its masking capabilities and the ability to exchange information with its external surroundings. The proposed approach goes beyond exploiting spatially variable intrinsic parameters and object-specific stealth device performance. The proposed devices’ homogeneous material parameters significantly contribute to achieving acoustic external cloaking devices.

In conclusion, the objective use of coordinate transformations enables the regulation of sound wave propagation and reflection properties by adjusting the shape of objects or structures in the acoustic field. This creates a powerful tool for researching and designing acoustic devices, improving the focus of the acoustic field, or controlling the interaction between sound waves and objects.

#### 2.2.2. Medium Conditioning

The coordinate transformation methods can be used to condition the medium of sound waves. As sound waves propagate in various media, their propagation characteristics often alter. Through the implementation of suitable coordinate transformations and equivalent media, the process of describing and mathematically calculating sound waves can be simplified. The adjustment of medium parameters in sound waves, including impedance distribution, medium density, and modulus, can be achieved through appropriate transformations and settings, enhancing the efficiency of the calculations while maintaining a certain degree of accuracy. The application of coordinate transformations and equivalent media facilitates the redefinition of medium properties in each section of the acoustic field, thereby simplifying and enhancing the problem’s explanation and solution. This technique is extensively utilized in acoustic simulation, the analysis of sound fields, and optimal design. It is crucial for both research and engineering purposes.

**Figure 3:**
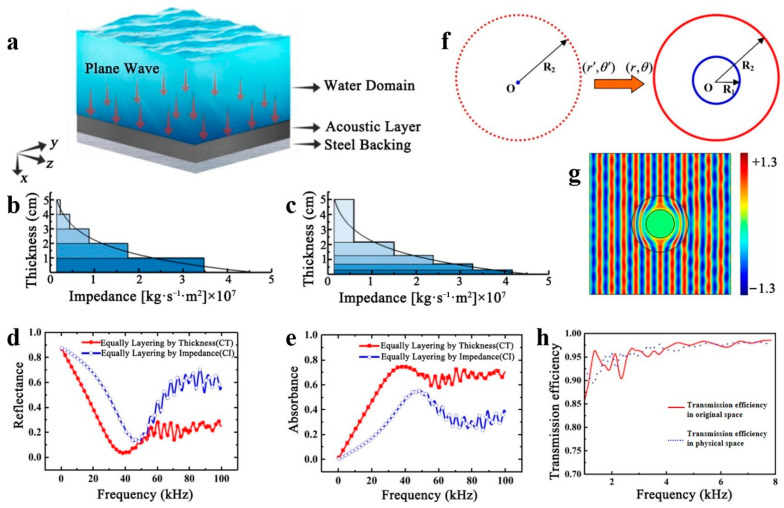
(**a**) Model diagram. Two layering methods: constant thickness layering (**b**) and constant impedance layering (**c**). The absorption coefficient (**d**) and reflection coefficient (**e**) simulated by the two methods. Reproduced with permission from [67]. Copyright © 2021, Elsevier. An acoustic cloak is designed based on a coordinate transformation method (**f**), which has good stealth performance (**g**) and high transmission efficiency (**h**). Reproduced with permission from [68]. Copyright © 2021, Springer Nature.

An important aspect of the medium parameters is the impedance distribution. Impedance denotes the ratio of sound pressure and sound flow at the interface of a medium when a sound wave encounters the interface during propagation. The impedance difference emerges as sound waves propagate in a medium and significantly influences the reflection and transmission behavior of sound waves at the interface. Therefore, the impedance distribution of the medium can regulate the reflection and transmission behavior of sound waves between different media. Y.W. Feng et al. conducted research on composite media with different gradient impedances, and their results show the effect that the shape of the impedance distribution has on the acoustic performance [67], as shown in Figure 3a by the absorption and reflection coefficients calculated using a geometric model. Methods for media distribution are shown in Figure 3b,c, with Figure 3b showing thickness contour (CT) stratification and Figure 3c revealing impedance contour (CI) stratification. The absorption and reflection coefficients obtained are displayed in Figure 3d,e. The two media distribution methods display distinct acoustic properties, with the CT method generally exhibiting lower reflection coefficients and higher absorption coefficients than the CI method. Therefore, the CT method leads to superior acoustic performance. When enhancing impedance matching, the typical approach is to streamline the material parameters, which can be accomplished through acoustic metamaterials. To achieve simplified parameters for impedance matching in acoustic design, J. Cao et al., concentrating on impedance matching techniques, proposed a method of impedance-adjustable transformation acoustics [68]. According to established theories of coordinate transformation, a substance is positioned within the initial space, and an appropriate coordinate transformation is executed to ascertain the transformation medium within the physical space. Impedance matching can be manipulated by adjusting the original space settings without altering coordinate transformations. In their research, J. Cao et al. examined impedance matching by combining original space settings with selected associative coordinate transformations. Figure 3f presents a two-dimensional coordinate transformation method that simplifies parameters by adjusting impedance distribution in the geometrical limit. The parameters of the transforming medium can be set using a combination of tunable impedance functions and appropriate linear or nonlinear coordinate transformations. Adjusting the impedance distribution simplifies the parameters. The fixed mass density ρ is employed, and the related coordinate transformations and impedance functions are achieved through coordinate transformation and impedance matching based on the boundary conditions. By adjusting the impedance settings in the original space and selecting corresponding coordinate transformations, it is possible to obtain multiple impedance-matched transformation media. The simulation of the pressure field revealed a near-perfect stealth performance, as illustrated in Figure 3g, while still maintaining a high transmission efficiency, as demonstrated in Figure 3h. This method can potentially be applied to other transformed acoustic designs, such as the three-dimensional case. 

**Figure 4:**
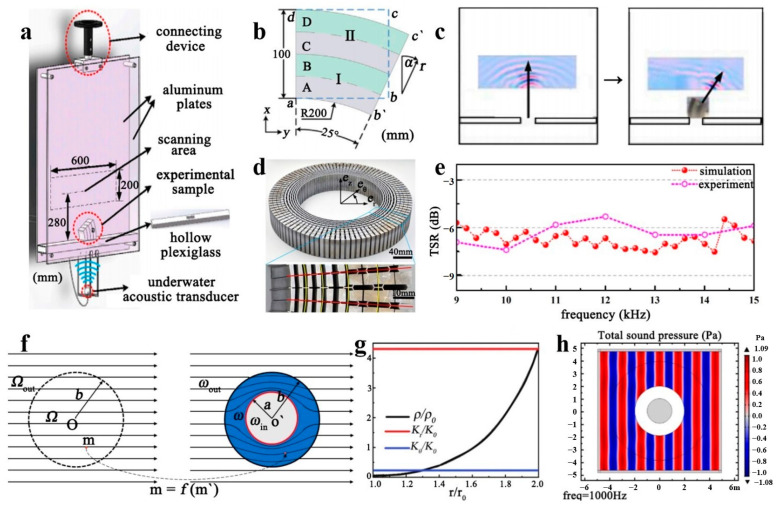
(**a**) Experimental device diagram. (**b**) The schematic diagram of the acoustic waveguide based on the coordinate transformation. (**c**) The experimental results of the acoustic waveguide at 20 kHz. Reproduced with permission from [75]. Copyright © 2022, Frontiers. (**d**) Acoustic cloak structure. (**e**) Simulation and experimental results of acoustic cloak stealth performance at 9–15 kHz. Reproduced with permission from [76]. Copyright © 2017, APS Publishing. (**f**) A coordinate transformation method. (**g**) Design the parameters of acoustic cloak materials by changing the density and modulus. (**h**) Stealth performance. Reproduced with permission from [77]. Copyright © 2022, MDPI Publishing.

Two commonly used medium models in coordinate transformation are the five-mode medium and the inertial medium. The inertial medium is usually used as a density anisotropic element fluid [69]. To realize the flexible manipulation of sound waves, it usually requires a large density anisotropy. At present, the density anisotropy that can be realized by the technical scheme is very small. Moreover, the inertial medium is affected by the material or the working frequency band, and the engineering significance is limited [70]. The five-mode medium is a three-dimensional solid mechanics metamaterial that is artificially manufactured to withstand only single-mode stress [71]. The five-mode medium has five zero eigenvalues in the sixth-order elastic matrix, which can be easily deformed in the corresponding five independent modes. For example, isotropic five-mode media are often used to mimic fluid behavior. Because under ideal conditions the bulk modulus is infinitely greater than the shear modulus [72], the isotropic five-mode medium is difficult to compress, but it flows easily. Compared with traditional inertial stealth, five-mode acoustic stealth can avoid mass singularity, and can be designed with pure solid materials, and is theoretically broadband. The five-mode medium enables linearization of the elastic wave equation and control of fluctuation behavior by coordinate transformation. The five-mode medium is utilized as a model with unique homogenization properties under coordinate transformation by selecting a specific geometric structure and material parameters. This flexible and scalable model allows for fine control of fluctuations, which is advantageous for examining and designing physical systems with wave control features [73,74]. Currently, the study of anisotropic modulus metamaterials through the application of five-mode metamaterials is prevalent. In contrast, the study of anisotropic density metamaterials through the application of five-mode metamaterials is less common. X. Chen and colleagues developed a technique for achieving anisotropic densities in fluid-like materials utilizing five-mode materials [75]. Figure 4a displays the experimental setup. The methodology used for the transformation of experimental samples is illustrated in Figure 4b. The rectangular area, abcd, denotes the spatial region before the transformation, whereas ab’c’d denotes the same region after the transformation. This method allows samples containing two or more distinct five-mode cellular structures to be assembled into waveguides with different deflection angles. At a frequency of 20 kHz, the results obtained from the measurement are displayed in Figure 4c, where sound waves propagate along the bends with the designed deflection angle. No noticeable scattering takes place around the path of sound wave propagation. This structural design enables the accurate and efficient manipulation of hydroacoustic waves. As achieving anisotropic density in water is challenging, transform acoustics in hydroacoustics typically utilizes the anisotropic modulus to a greater extent. Y. Chen et al. proposed a solid medium based on a five-mode material for the first time to achieve an underwater stealth cloak [76]. The structure of this cloak is illustrated in Figure 4d. It has been experimentally verified that the stealth cloak has excellent target strength reduction (TSR) performance in the broadband frequency range of 9–15 kHz, and can achieve a good stealth effect, as shown in Figure 4e. This discovery sets the stage for applying structural anisotropic modulus superfluid control of underwater acoustics. However, in light of the correlation between the density and modulus of the five-mode material and the acoustic cloaking cloak’s parameter variation across different layers, it is imperative to conduct extensive structural optimization work on the five-mode material to satisfy the specific parameter prerequisites. Researchers have investigated five-mode material configurations to simplify the design process, independently transforming the density and modulus. The acoustic stealth cloak design can be completed using material-specific structure-matching constant mode mapping coordinate transformation equations. Refer to Figure 4f for a visual representation [77]. The coordinate transformation equations and variation rules for the material parameters of the equal-mode loudspeaker concerning radius are shown in Figure 4g. The equal-mode cloak exhibits a favorable transmission effect on incident sound pressure, with minimal disturbance to the background sound pressure field, as depicted in Figure 4h. The cavity covered by the acoustic cloaking cover with a radius of 0.5 m has an average total scattering cross-section of 0.858. This was determined by mapping the boundaries obtained through simulation calculations and optimized design in the range of *a*/*λ* from 0 to 1. Upon removing the five-mode material acoustic cloaking cloak, the cavity’s average total scattering cross-section is 19.718. The study results are expected to provide methodological guidance for streamlining the design of acoustic stealth cloaks using five-mode materials and their microstructural design. Nonetheless, the development of acoustic cloaks for actual submarines faces substantial technological challenges for underwater applications that remain unfulfilled. These challenges include handling weight or non-axisymmetric shapes. Alternatively, elliptical coordinates may be utilized to define quasi-symmetric transformations to extract the material characteristics of the five-mode cloak for ellipsoidal targets alongside measurable approximations resulting from rotation tensors distinct from the constant ones. Regarding the previous methods of dealing with arbitrarily shaped five-mode cloaks, this technique enables control of the dominant direction of anisotropy beforehand. It broadens the design possibilities by exploring various material property distributions for the same geometrical problem.

**Figure 5:**
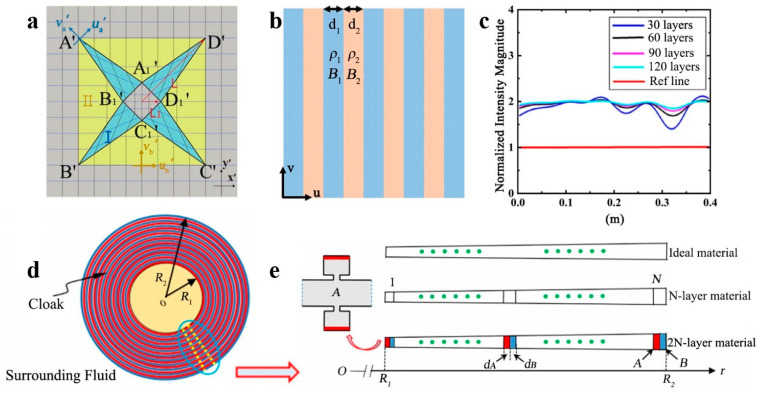
(**a**) A coordinate transformation method. (**b**) Layered structure with different density and bulk modulus. (**c**) Scattering results of acoustic waves under different layers. Reproduced with permission from [78]. Copyright © 2021, Springer Nature. (**d**,**e**) The design process of the acoustic cloak. Reproduced with permission from [79]. Copyright © 2019, Elsevier.

As a complex and adaptable medium, multi-layered effective media can have a variety of physical properties, including isotropy, anisotropy, negative refraction, and others. By precisely designing the combination, thickness, and arrangement of various material layers, the control and regulation of fluctuations in multi-layered effective media can be achieved, presenting a broad range of potential applications. The design of such media can be simplified through linear coordinate transformation [78]. Dividing the device into polygonal subregions can achieve linearity, as illustrated in Figure 5a. The effective medium theory enables the multilayer structure’s design through various materials, as depicted in Figure 5b. The performance of the device design is confirmed through numerical simulation, as shown in Figure 5c, with the results demonstrating good agreement with the theoretical predictions. This approach could be extended to other acoustic devices with applications in diverse fields, such as medical ultrasound therapy and underwater communication. N. Li and colleagues examine the topics of underwater stealth and piezoelectric sheet design [79,80]. They introduce a novel class of active elastic metals and stealth designs incorporating dual Helmholtz cavities. By varying the applied voltage and the stiffness of the various piezoelectric layers, the structure can be made to exhibit a controllable effective bulk modulus with negative values over a wide range of frequencies. The parameters for the multilayer acoustic cloak can be acquired through the coordinate transformation method and corresponding effective medium. This effect is attainable through active control of each unit cell to modify the dispersion of the effective bulk modulus, and the design of the structure is displayed in Figure 5d,e. These characteristics offer a wide frequency range for adjusting the effective bulk modulus, a crucial aspect of controlling elastic wave propagation. N. Li et al. developed an active elastic metamaterial featuring a cavity and an acoustic concentrator. The piezoelectric diaphragm’s feedback-controlled gain can provide an adjustable effective bulk modulus and density across a broad frequency spectrum. Based on the coordinate transformation method, employing feedback control in the acoustic cavity periodical arrangement enables the achievement of multilayer optional homogeneous materials for the polycondenser. Numerical calculations demonstrate that the acoustic waves are utterly confined within the concentrator’s core region, thereby augmenting the acoustic energy. The above-mentioned characteristics, coupled with a wide range of tunability in effective bulk modulus and density, are projected to aid in developing functional acoustic concentrators.

In summary, coordinate transformations are important in regulating electromagnetic and acoustic waves in relation to the division of objects into multiple regions and medium regulation. The coordinate transformation methods can address the multi-region division of objects in the field and adjust the shape of objects or structures by means of coordinate transformation. Through suitable coordinate transformations, it is possible to modify the shape or structure of an object, which consequently influences the characteristics of wave propagation and reflection. Additionally, these transformations can assist in handling complex boundary conditions by simplifying them or introducing equivalent forms. Furthermore, it is feasible to utilize coordinate transformations for medium conditioning to adjust field parameters by introducing an equivalent medium.

## 3. The Acoustic Properties of Sound Waves Are Influenced by the Regulation of Polymer Acoustic Metamaterials

In recent years, acoustic metamaterials have emerged as prominent topics in acoustic manipulation. The place of acoustic metamaterials in the acoustics field underlines their importance as means and tools for acoustic control and manipulation. By artificially designing materials and structures, acoustic metamaterials can accurately control the propagation characteristics of sound waves. Polymers have shown a significant role in the field of acoustic metamaterials. According to the requirements of acoustic applications, polymer metamaterials can be flexibly designed to significantly improve the propagation characteristics of sound waves by optimizing their structure and morphology. It is helpful to develop new acoustic sensors, efficient sound absorption equipment, and high-performance acoustic waveguide devices. The following is a summary of how acoustic metamaterials modulate the frequency, the phase, and the scattering of acoustic waves.

### 3.1. Frequency Manipulation

**Figure 6:**
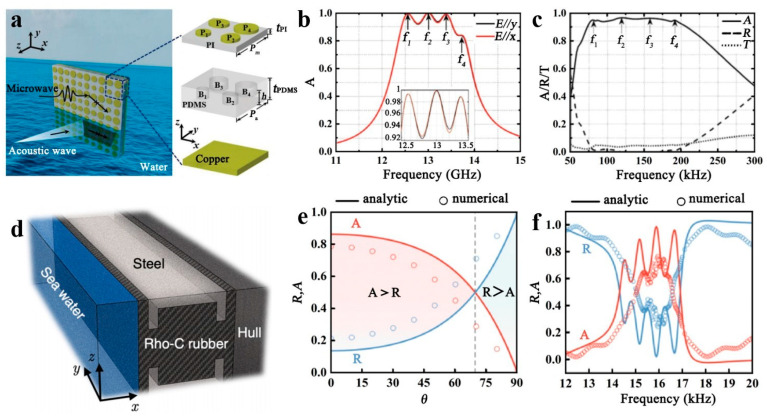
(**a**) Structure of electromagnetic acoustic metamaterials. (**b**) Free-space microwave absorption (A) properties. (**c**) Water acoustic wave absorption (A), reflection (R), and transmission (T) properties. Reproduced with permission from [6]. Copyright © 2020, Wiley. (**d**) Metamaterials’ structures based on steel and rubber. (**e**) The variation in absorption (A) coefficient and reflection (R) coefficient with frequency in single-layer metamaterials. (**f**) The variation in absorption (A) coefficient and reflection (R) coefficient of multilayer metamaterials with frequency. Reproduced with permission from [82]. Copyright © 2021, AIP Publishing.

Frequency manipulation is a significant area of research in acoustics, particularly in acoustic stealth, which is closely associated with frequency manipulation. G.S. Sharma and colleagues investigated the acoustic absorption properties of underwater viscoelastic coatings with periodically distributed voids and hard inclusions [81]. The team approximated each layer of the grid as a homogeneous medium with effective material and geometrical properties using effective medium theory. The study considered the unipolar and dipolar resonances of the inclusions. Finite element modeling was developed to study periodically distributed hollow and hard inclusions in a rubber medium. Different combinations of layers showed significant variations in acoustic performance, with the water liner or steel liner significantly affecting the acoustic performance of the different inclusions. Soft elastic media such as rubber, with an impedance close to that of water, were used to prevent acoustic reflections. In addition, soft elastic media embedded in inclusions induce local resonance. This enhances sound dissipation by promoting the conversion of longitudinal waves into transverse waves. In engineering, differences may arise between acoustic metamaterials placed on various shell surface areas, including exposed ones. Due to the complex nature of the acoustic metamaterial layout, quantitative computational methods must be developed to evaluate the impact of acoustic coatings on the reduction of underwater acoustic radiation. Y. Zhou and colleagues proposed an ultrathin electromagnetic acoustic amphibious stealth coating [6]. This technology is expected to have broad application in achieving stealth capabilities for aquatic objects. The resonance of the bubble metascreen in the copper plate substrate PDMS is used for hydroacoustic sound absorption. The combination of four bubble planes of different diameters into a single super monolith is shown in Figure 6a and can greatly expand the operating bandwidth. With the controlled thickness of the components, the key idea of dual-physical absorption with dual-impedance matching between air and water can be realized. It has been confirmed through simulation and experimentation that free-space microwave absorption rates exceed 90% within the range of 12.38–13.7 GHz. Moreover, water acoustic wave absorption rates surpass 90% within the frequency range of 72–208 kHz and more than 80% in the range of 65–232 kHz, as demonstrated in Figure 6b,c. These results showcase an innovative stealth technology that can tackle free-space microwaves and underwater acoustic waves, opening up numerous practical applications. D. Lee and colleagues proposed an underwater stealthy metamaterial that reduces the acoustic reflectivity of a split-aperture tube (SOC) hybrid resonator by embedding it into a constituent unit in Rho-C rubber [82], as shown in Figure 6d. The development of underwater acoustic absorbers is important for protection against sonar. Military submarines encompass a two-way system comprising mounting sonar and hydrophone receivers. In situations where the receiver is in a distinct coordinate system compared to other opponent vessels, it is crucial to consider the oblique incident waves transmitted through reflection to the coordinates outlined in Figure 6e. The efficiency of the absorption gradually decreases as θ increases.

However, it remains satisfactory. To continue our study of hybrid acoustic metamaterials with different fundamental frequencies, we now focus on their coupling capabilities to achieve enhanced absorption, as shown in Figure 6f. In addition, the absorption capabilities of these hybrid acoustic metamaterials remain sustainable over a range of *θ* values. This mechanism, allowing for easy resonance coupling, makes it possible to obtain broadband absorption spectra even in deep ocean conditions, despite the presence of covariant sound velocity profiles and thermo-viscous effects in the metamaterial unit cells. Variations in sound velocity caused by changes in temperature, salinity, and seawater depth, or thermo-viscous effects on the rigid boundaries of the metamaterials, have a negligible impact on the absorption spectra. These underwater metamaterials can adhere to curved surfaces. They can be used directly as a next-generation anechoic coating for submarines.

### 3.2. Phase Manipulation

**Figure 7:**
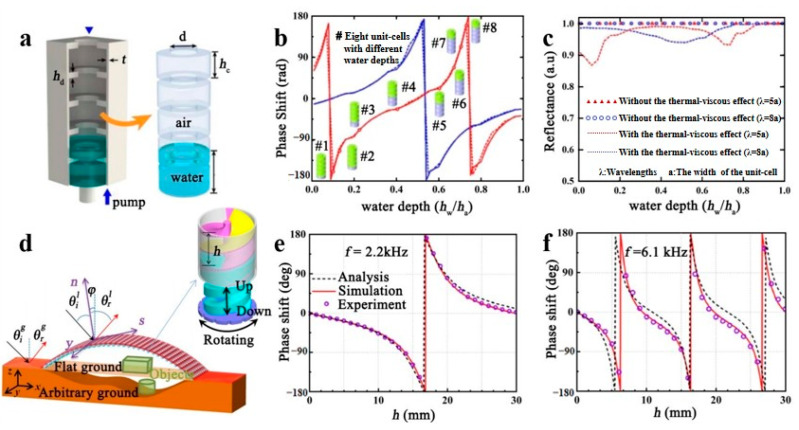
(**a**) The structure of pumping water into the metamaterials. (**b**) The relationship between the change in water depth and the phase shift in the structure, and (**c**) the reflection effect of the metamaterials on the sound wave at different water depths. Reproduced with permission from [83]. Copyright © 2020, IOP Publishing. (**d**) Metamaterials with helical structure. The acoustic phase changes with the spiral depth at 2.2 kHz (**e**) and 6.1 kHz (**f**). Reproduced with permission from [84]. Copyright © 2020, APS Publishing.

**Figure 8:**
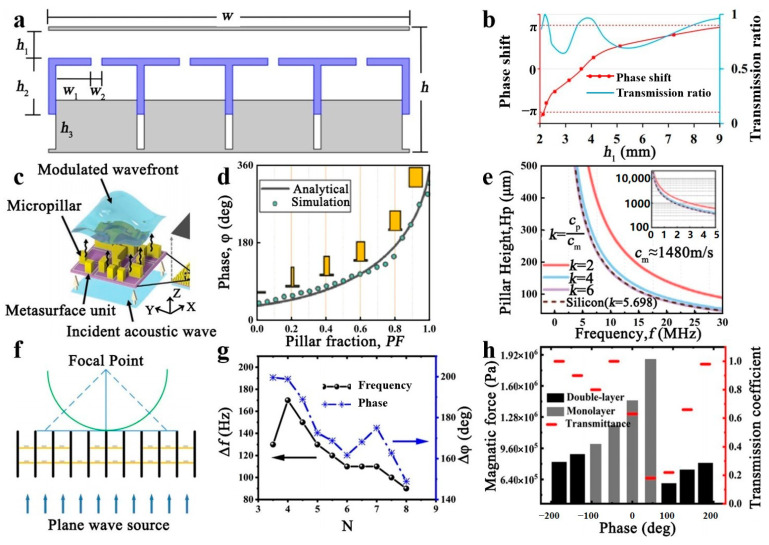
(**a**) Metamaterials based on structural movement. (**b**) The relationship between the change in h1 in the structure and the phase shift and the transmission ratio. Reproduced with permission from [85]. Copyright © 2019, IOP Publishing. (**c**) Metamaterials based on surface micropillars. (**d**) The relationship between the change in the micropillar and the phase shift in the structure. (**e**) The height of the micropillar decreases with the increase in frequency and metamaterials’ velocity. Reproduced with permission from [86]. Copyright © 2023, Wiley. (**f**) Metamaterials’ structure under magnetic force. (**g**) The effect of film spacing on frequency and phase changes in the structure. (**h**) Phase distribution and transmission coefficient at different membrane tensions. Reproduced with permission from [87]. Copyright © 2020, AIP Publishing.

Phase manipulation plays a significant role in the manipulation of acoustic waves. Several methods can be used to control the phase of acoustic waves, including modifying the wave propagation path, using interference effects, and implementing wavefront manipulation techniques. X.S. Li et al. studied a flexible curved surface made up of a unit cell with corrugated holes [83]. As illustrated in Figure 7a, water is introduced into the unit cell through a pump located at the bottom. Due to the significant impedance mismatch between air and water, the phase delay of the incoming acoustic wave can be adjusted by changing the water depth hw entering and exiting the hole. In Figure 7b, the phase shift is presented for various operating wavelengths depicted by the red and blue lines, respectively, as a function of water depth. The phase covers [0,2π] and fulfills the complete acoustic wave control demand. Figure 7c demonstrates that the incident wave’s energy is entirely reflected in the absence of losses. However, with the introduction of the thermo-viscous effect, the reflectivity remains relatively constant, except for a sudden shift in the phase profile at the depth of the water. Overall, the reflectivity exceeds 0.85, and no changes significantly affect the performance of the acoustic metamaterials. By adjusting the water depth distribution, these designed metamaterials can achieve anomalous reflection, focusing, and ground illusion over a wide frequency range. Numerical simulations confirm the feasibility of this design. Compared to planar reflectors, these curved reflectors expand the potential applications of reflective metamaterials. S.W. Fan and colleagues developed a spiral cell junction that can be adjusted [84], as depicted in Figure 7d. The adjustable cell was then utilized to create curved metamaterials that either recovered or mimicked the reflected waveform. All the samples were PLA. The theoretical, numerical, and experimental investigations of the channel length, which is indicated by the spiral depth, are illustrated in Figure 7e,f. This study evaluated the continuous full 2π phase shift across the frequency range of 2–7 kHz. As a practical example, this paper presents the concept of curved acoustic metamaterials for continuous tunable acoustic multifunctionality, including broadband carpet cloaking and ground illusions across a wide operating band. Full-wave numerical simulations are employed to demonstrate this concept. Z.C. and colleagues developed a mobile slider element structure [85], shown in Figure 8a, which uses four lumped Helmholtz resonators for phase control. The design changes the resonant characteristics by changing the slider’s position. This allows the system to be partially fixed and controlled without changing the overall dimensions of the structure. The main benefit of this design is that it permits the whole structure to be made of one material, offering the possibility to alter the unit dynamically while keeping the system stable. The metamaterial unit is prepared by 3D-printed polymethyl methacrylate. The devised metamaterial unit covers a phase of 2π with noteworthy transmission efficiency, as shown in Figure 8b. The metamaterial unit created by M.X. Xu and the team consists of micropillars [86], where the phase shift of each metamaterial unit is determined by the volume fraction of the micropillar and is adjusted by varying the phase of the acoustic wave transmitted through the metamaterials as shown in Figure 8c. Moreover, the height of the column is less affected by the ratio of sound velocity, and it is negatively correlated with frequency. Consequently, increasing the frequency does not necessitate a high metamaterial height, as depicted in Figure 8d,e. Therefore, this type of metamaterial is particularly suitable for high-frequency (>5 MHz) applications. Acoustic holography is used to verify the flexibility of metamaterials, in which PDMS particles are used to visualize the sound field. The unique phase diagram of metamaterials leads to arbitrarily defined amplitude distribution on the target plane. P. Liu and colleagues investigated a magnetically controlled approach to achieve multifunctional acoustic metamaterials by merging structural design and magnetic field application [87]. The magnetic force can continuously adjust the properties of the acoustic metamaterials, which consist of an elastic membrane and additional mass, as shown in Figure 8f. The phase range can be shifted from −π to π by adding single or double membranes, as shown in Figure 8g. Figure 8h illustrates distinct magnetic and transmission values aligned with different phases observed at 985 Hz. The other magnetic forces resulting from the different phase requirements can be adjusted, and phase switching is also possible. By modulating the magnetic force, the transmitted acoustic waves can be readily modified, enabling various functions like focusing, similar beam splitting, and other near-field acoustic displays to be switched. This work expands on the research of multifunctional metamaterials with great potential for various applications, such as acoustic imaging, communication, and particle manipulation (e.g., levitation and acoustic tweezers).

### 3.3. Scatter Manipulation

Scattering manipulation is essential in modulating acoustic waves. In simple terms, it allows acoustic waves to be controlled. This is achieved by controlling the scattering characteristics of the surface of a material or structure.

**Figure 9:**
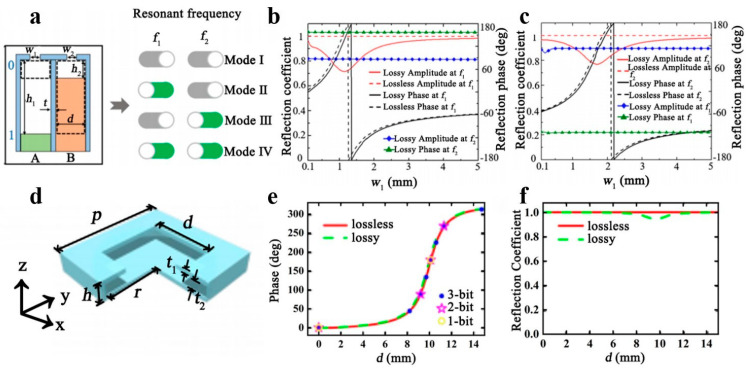
(**a**) The structure of the coding metasurface. The relationship between the reflection phase, the reflection coefficient, and the neck width *w*_1_ (**b**) and *w*_2_ (**c**) of the two subunits at *f*_1_ and *f*_2_. Reproduced with permission from [88]. Copyright © 2021, Elsevier. (**d**) The structure of the coding metasurface unit. The reflection phase (**e**) and reflection coefficient (**f**) change with the length d of the neck side in the structure. Reproduced with permission from [89]. Copyright © 2019, AIP Publishing.

Since digital circuits have only two states, “ON” and “OFF,” the rapid development of coded control has inspired various fields. Acoustic metasurfaces are proposed to encode meta-atoms as “0” and “1”. Acoustic scattering regulation can be effectively achieved by designing the coding sequence in advance. X.P. Song et al. reported a 2-bit coded acoustic metasurface technique combined with the structure of a Helmholtz resonant cavity [88]. A 2-bit coding element atom is utilized, comprising two Helmholtz resonator-like subunits labeled A and B, each representing “0” or “1”. By altering the position of the blocks within the cavity, four modes of coding can be achieved: 00, 01, 10, and 11, as demonstrated in Figure 9a. This configuration allows for levitation and frequency manipulation. Figure 9b,c illustrate the meta-atomic reflection angles and coefficients concerning the neck widths *w*_1_ and *w*_2_ of the two substituents at *f*_1_ and *f*_2_. Despite the losses taken into account during the simulation, the reflection coefficients remain high, and the reflection phases cover almost the entire range of 2π. The encoding mode of the meta-atoms can be altered to produce different negative and specular reflection properties at different frequencies by shifting the block’s position within the subunit. Independent manipulation of the oblique incident wave can be accomplished by manipulating the parameters of the subunits at their specific resonant frequencies. By regulating the number of outgoing diffracted waves to extract the higher-order diffraction modes, the undesirable sidelobes can be eradicated, leading to a completely negative reflection. Generally, achieving negative reflection from an acoustic metasurface is governed by the generalized Snell’s law. Still, the efficiency is strongly affected by the angle of incidence, with high efficiency only at low angles of incidence. This study overcomes this limitation and achieves perfect negative reflection. The coded acoustic metasurface design method is simple enough to apply to multifunctional, frequency-dependent, and programmable acoustic devices. In addition, most coded acoustic metasurface designs have only two encoding units, which provides greater control over the acoustic wave behavior but limits functionality. A multi-bit encoded acoustic metasurface has been proposed by Y. Zhang et al. [89]. The metasurface can hold eight or more coding modes and is constructed as a Helmholtz resonator with varying geometrical parameters. The coding metamaterial sample is shown in Figure 9d, and the coding metasurface sample is coated with PLA using 3D printing technology. Figure 9e,f display the change in reflection phase and reflection coefficient when the length of the neck side of the Helmholtz resonator is varied at a frequency of 7 kHz for both lossless and lossy cases. The 3-bit coded metasurfaces undergo digital convolution, affirming their capability to generate and modulate acoustic vortex beams. Additionally, the thermal viscosity effect has minimal influence on the multi-bit encoded acoustic metasurface. This enhances the regulation of acoustic waves with greater efficiency and flexibility, thus holding immense promise for practical applications. In hydroacoustic wave applications, Y. Liu et al. produced aqueous acoustic metamaterials inspired by natural superhydrophobic systems [90]. The proposed acoustic metamaterials feature a laser-textured superhydrophobic cavity structure, seamlessly connecting the laser-prepared superpositioned layer with the aluminum alloy plate substrate. Through acoustic tests, it has been determined that the acoustic metamaterials’ design demonstrates excellent performance within the frequency range of 50–60 kHz, displaying exceptional sound isolation and a significantly decreased water-acoustic transmission coefficient. This effectively controls low-frequency sound waves underwater and provides essential technical support for underwater acoustic stealth. Y. Bai and colleagues investigated the remote manipulation of hydroacoustic waves using an acoustic superscatterer [91]. This device consists of an acoustic coating and an inner core, which can be placed together at a specific location to achieve effective omnidirectional radiation mitigation. The design has great potential for studying and developing remote meta-device manipulation of hydroacoustic waves with sources of the same cross-sectional shape.

**Figure 10:**
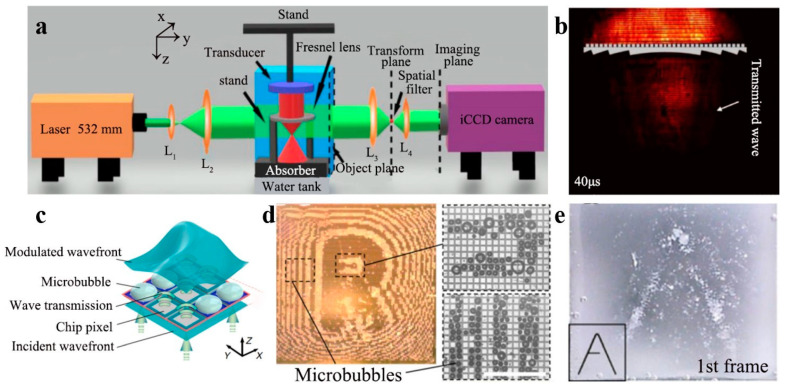
(**a**) The schematic of the experimental device. (**b**) The sound field real shot of sound wave through the Nefer lens. Reproduced with permission from [92]. Copyright © 2020, IOP Publishing. (**c**) The structure of bubble-based modulator, and imaging results (**d**,**e**). Reproduced with permission from [93]. Copyright © 2020, Springer Nature.

In the medical field, Q.X. Zhou et al. proposed a high-efficiency ultrasonic Fresnel lens submerged in water. In the study [92], the acoustic pressure gain of the Fresnel lens with and without a grating on the back was investigated using the experimental setup shown in Figure 10a. It was found that the transmission coefficient of the incident acoustic wave through the lens equipped with a grating on the backside exceeded that of the lens without a grating, as shown in Figure 10b. Further refinement of the grating width in the structure could significantly increase the acoustic pressure gain. The lens has potential applications in acoustic imaging and medical diagnostics. Z.C. Ma et al. presented a dynamic spatial ultrasound modulator with the unit structure illustrated in Figure 10c [93]. It can dynamically reshape an incident plane wave into a complex acoustic image. The transfer function of the apparatus is determined by a microbubble pattern generated digitally and regulated by a complementary metal–oxide–semiconductor (CMOS) chip. This configuration generates binary amplitude acoustic holograms, as depicted in Figure 10d,e. In order to visualize the sound pressure field on the target plane, sub-millimeter-scale PDMS particles suspended in water are introduced and then combined into the shape of the projected sound pressure image. The spatial ultrasound modulator enhances the efficacy of ultrasound techniques and offers considerable potential for holographic acoustic tweezers. Holographic acoustic tweezers could be used in more complex procedures where particles or cells captured by manipulation have various roles, including holding, release, orientation, separation, or aggregation.

**Figure 11:**
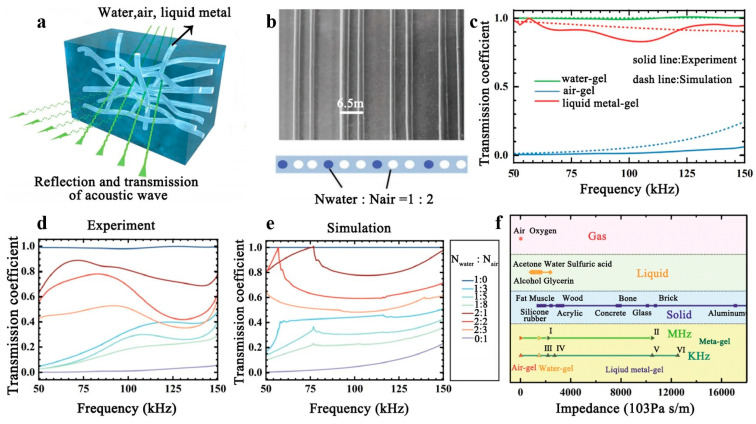
(**a**) Metagel filled with different media. (**b**) Filling different media proportionally in parallel channels. (**c**) The acoustic transmission coefficient of the metagel when filled with water, air, and liquid metal. The acoustic transmission coefficient (**d**) of the metagel when filled with water and air in different proportions. Experiment: (**e**). Simulation: (**f**). Adjusting the filling medium, the acoustic impedance of the metagel can achieve a wide range of matching. Reproduced with permission from [20].

Recently, it has been discovered that hydrogels have significant potential for the manipulation of the scattering of acoustic waves. Hydrogel is a polymer network system with a hydrophilic three-dimensional mesh crosslinked structure that is soft in nature, can maintain a specific shape, and can significantly absorb water. Its exceptional properties have gained considerable attention and have been widely used in numerous fields, including medicine for drug delivery and tissue regeneration, due to its high biocompatibility. Researchers have recently devoted their efforts to studying the acoustic properties of hydrogels [94,95,96]. C.W. Zhang and colleagues proposed a soft robotic fish inspired by manta rays [97], whose structural element is made of a tough hydrogel and powered by a dielectric elastomer. This design enables the robotic fish to swim stably while exhibiting excellent acoustic stealth performance. The robust hydrogel fish contains a significant amount of water and has an acoustic impedance comparable to that of certain marine organisms. Sound waves passing through the hydrogel fish show no detectable interface, indicating its excellent acoustic stealth performance in marine environments. Recently, K. Zhang et al. introduced metagel, hydrogel composites that can be tuned to have adjustable acoustic properties across a broad frequency range [20]. The channels are embedded within a hard hydrogel matrix. The filler medium, including air, water, or liquid metal, is incorporated as required to adjust the acoustic wave transmission of the metagel, depicted in Figure 11a. It is assumed that the acoustic wave is nearly fully propagated when the channel is filled with water. When the channel is filled with air, the acoustic properties of the metagel can approximate those of air to enable complete reflection of the acoustic wave. When the channel is filled with liquid metal, the acoustic properties of the metagel can approximate those of soft solid materials, such as bio-tissues and engineered rubbers, to achieve the dual roles of acoustic wave transmission and reflection. In the report, the researchers propose two primary approaches to modify the acoustic properties of the metagel: first, by changing the filling substance in the channel; second, by varying the filling ratio of different substances, as shown in Figure 11b. The first method is to adjust the acoustic performance of the channel by changing the filling material used in the channel. After conducting simulations and experiments, it has been determined that sound wave propagation is almost optimal in the frequency range of 50~150 kHz when the channel is filled with water. Conversely, the sound wave is almost entirely reflected when the channel is filled with air. When liquid metal fills the channel, the average transmission coefficient measures approximately 0.9. As demonstrated in Figure 11c, these findings suggest that the metagel possesses an adaptable hydroacoustic transmission coefficient within the frequency range of 50~150 kHz, dependent on the variety of filling substances. In the second approach, the filling ratio variation, the channel diameter values, and the distance between neighboring channels are kept constant. In contrast, the filling ratio of the water and air channels is varied. The acoustic transmission coefficient curves were charted based on the experiments in the 50–150 kHz range. The results are depicted in Figure 11d,e. By adjusting the filling ratio, it was found that the transmission coefficient could cover the entire range from 0 to 1. In addition, the acoustic properties of the metagel were investigated at MHz frequencies using an ultrasound imaging system. It was observed that pumping water or air in the appropriate area could improve the contrast of ultrasound imaging, enabling the “on” and “off” imaging windows of the target object to be realized on demand. Finally, the acoustic impedance values of different metagel sizes and fillings are plotted in Figure 11f. By adjusting the filling medium, the acoustic impedance values can cover typical liquids and soft solids, even comparable to gases. Furthermore, if the wavelength of the acoustic wave in water exceeds the characteristic size of the metagel sheet, the acoustic impedance tunability is determined by the material composition rather than the resonance, allowing the metagel to function across a broad frequency spectrum. This discovery paves the way for future revolutionary design and application of acoustic materials.

Acoustic metamaterials are the frontier field of acoustic wave control developed in recent years. They allow the specific control of acoustic waves using precisely designed and arranged microstructures. The application of polymers in the field of acoustic metamaterials is becoming more and more significant. Polymers can design density and structure, and their diversity and plasticity make them an ideal choice for designing acoustic metamaterials. They can be combined with the coordinate transformation method, which promises more precise and efficient manipulation of acoustic properties. Coordinate transformation methods can be used to design acoustic metamaterials by mathematically and computationally optimizing the geometry and structure of the materials to meet specific needs and objectives for the propagation and transmission of acoustic waves. The coordinate transformation methods may be utilized in designing acoustic metamaterials. These methods involve controlling acoustic waves’ interference and scattering features by transforming the coordinates of the acoustic metamaterials. In conclusion, integrating acoustic metamaterials with topology optimization and the coordinate transformation approach offers a feasible and precise means of controlling acoustic waves, leading to new advances and growth opportunities in acoustic applications.

## 4. Conclusions and Prospectives

Employing coordinate transformations enables the spatial transformation and deformation of acoustic waves, altering their propagation path and characteristics. The microstructure of polymer acoustic metamaterials can be meticulously designed and controlled, allowing for the manipulation of acoustic properties such as frequency and phase scattering. However, challenges may arise when using coordinate transformation methods and acoustic metamaterials to modulate acoustic waves. Coordinate transformation methods are unique techniques that can direct, concentrate, and manipulate acoustic waves by modifying the spatial coordinate system’s transformation relationship. Acoustic metamaterials are engineered to precisely control the propagation and response of acoustic waves by adjusting their microstructure. The integration of coordinate transformation with acoustic metamaterials holds significant potential for effective acoustic wave manipulation. At present, polymer acoustic metamaterials face many obstacles in acoustic manipulation. Coordinate transformation methods can provide strong support, but there are still challenges that need to be further studied and solved:

(1) Structural design issues: The precise manipulation function of acoustic waves depends on the structural design of polymer acoustic metamaterials. By combining the structural transformation capabilities of the coordinate transformation methods, polymer metamaterials’ structures can be designed and optimized with specific shapes, periodicities, and geometrical parameters to achieve precise control of acoustic waves. Such designs must be optimized to achieve the desired results.
(2) Medium tuning issues: The properties of polymer acoustic metamaterials are affected by the material parameters, so tuning the medium parameters can change the acoustic properties and enable more precise tuning of acoustic waves. Using the coordinate transformation methods’ medium adjustment capability, it is possible to design and adjust the medium parameters of polymer acoustic metamaterials, such as acoustic impedance, density, modulus, and acoustic transmittance. This allows frequency selectivity and phase manipulation of acoustic waves to be achieved.
(3) Practical application issues: The real use environment of polymer acoustic metamaterials is usually complex and changeable. The long-term stability, durability and performance in the complex environments of polymers need to be considered comprehensively. The future development direction needs to focus on improving the frequency response range of polymers, optimizing their sound absorption and acoustic control efficiency, and exploring new functional applications to promote the wide application and commercial development of polymer acoustic metamaterials.

In conclusion, the coordinate transformation methods can deal with the challenges of polymer acoustic metamaterials in structural design and medium adjustment. These methods integrate the capabilities of structural transformation and medium adjustment. With further research and advancements in the coordinate transformation methods, more complex and diverse polymer acoustic metamaterial structures can be developed to achieve extensive acoustic wave control functions. These methods offer a powerful tool for precise acoustic wave control and various acoustic applications, holding significant promise for the future. It will substantially advance innovation and applications in acoustics, including acoustic stealth, imaging, and sensing, leading to new breakthroughs in acoustic engineering and scientific research.

## Data Availability

No data were used for the research described in the Review article.
